# Exploring the Mechanism Whereby Sinensetin Delays the Progression of Pulmonary Fibrosis Based on Network Pharmacology and Pulmonary Fibrosis Models

**DOI:** 10.3389/fphar.2021.693061

**Published:** 2021-06-18

**Authors:** Yong Xu, Wen-Lu Hang, Xian-Mei Zhou, Qi Wu

**Affiliations:** ^1^ Affiliated Hospital of Nanjing University of Chinese Medicine, Nanjing, China; ^2^ Department of Respiratory Medicine, Jiangsu Province Hospital of Chinese Medicine, Nanjing, China; ^3^ Department of Respiratory and Critical Care Medicine, Second Affiliated Hospital of Xuzhou Medical University, Xuzhou, China; ^4^ Department of Physiology, Xuzhou Medical University, Xuzhou, China

**Keywords:** pulmonary fibrosis, sinensetin, network pharmacology, molecular docking, PI3K-AKT

## Abstract

The incidence of pulmonary fibrosis (PF), a progressively fatal disease, has increased in recent years. However, there are no effective medicines available. Previous results have shown that sinensetin probably has some curative effects on PF. Therefore, this paper aims to predict the targets of sinensetin using a network pharmacology method and to confirm its effects and functional targets in PF using a mouse PF model. First, network pharmacology analysis showed that sinensetin has 105 functional targets, and 1,698 gene targets closely relate to PF. The intersection of the functional targets and gene targets produced 52 targets for the treatment of PF with sinensetin. The PPIs (protein–protein interactions) led to several potential key target genes, including MAPK1, EGFR, SRC, and PTGS2. The results of GO and KEGG analyses suggested the crucial function of apoptosis in PF and its involvement in the PI3K signaling pathway. Subsequently, we tested the molecular docking of sinensetin with the PI3K protein using the AutoDock4 software. The results showed that sinensetin could fit well into several binding sites of the PI3K protein. Furthermore, we constructed a PF mouse model through one-off intratracheal instillation of bleomycin and then intragastrically administered different concentrations of sinensetin to the model mice. Twenty-eight days later, the mice were sacrificed, and the lung tissues, serum, and bronchoalveolar lavage fluid (BALF) were collected. The *in vivo* tests showed that the body weight of model mice increased slightly compared with that of PF mice after intragastric sinensetin. HE and Masson staining suggested a certain extent of reduction in the pathology of lung tissues. The expression of collagens I and III, as well as hydroxyproline in the lung tissues, was reduced to a certain extent. IL-6 levels in the serum and BALF decreased markedly. The expression of vimentin and *α*-SMA in pulmonary tissues decreased. Cell apoptosis, as well as P-PI3K and P-AKT levels, in lung tissues also reduced. In summary, network pharmacology and *in vivo* test results suggest sinensetin causes an effective delay in the progression of pulmonary fibrosis, and the functional mechanism is likely related to PI3K-AKT signaling.

## Introduction

Pulmonary fibrosis (PF) is a terminal lung disease featured by pulmonary fibroblast proliferation and extracellular matrix aggregation, often leading to abnormal lung tissue remodeling, respiratory failure, and even death (Kalluri et al., 2020). In recent years, the incidence of PF keeps increasing; patients generally have a poor prognosis, and no specific medicine is available for treatment. Thus, PF is emerging as a disease of global concern (Albert and Schwartz, 2019). Previously, PF was treated with glucocorticoids, but glucocorticoids have poor curative effects and severe side effects. Pirfenidone and nintedanib can only delay the rate of lung function decline in patients with moderate PF. In addition, these two drugs are expensive and induce notable adverse reactions (Kolb et al., 2018; Holtze et al., 2020). Our meta-analysis showed that Chinese medicines or a combination of Chinese and western medicines had a better effect in reducing the onset of acute fibrosis, improving the patient’s quality of life, and reducing the mortality of patients with idiopathic pulmonary fibrosis (IPF) compared with western medicine alone (Wu et al., 2019a). Therefore, we consider it possible to develop anti-fibrosis medicines based on traditional Chinese medicines.

Our previous study indicated that *Citrus reticulata* and its bioactive ingredients can reduce PF. We previously obtained citrus alkaline extract (CAE) by boiling it in alcohol, precipitation in an alkaline solution, and extraction in organic solvents. The *in vivo*/*in vitro* tests suggested that CAE could improve the pathology, inhibit collagen deposition in the lung tissues, reduce the transcript levels of inflammatory factors, and induce primary fibroblast apoptosis in mice with PF (Wu et al., 2018; Wu et al., 2019b). To further determine the effective ingredients in CAE, we examined five monomers, including sinensetin, which were likely to be the main components in CAE by UHPLC-ESI/LTQ-Orbitrap-MS and HPLC-UV analyses (Feng et al., 2019). After sequential filtration of these five monomers, we found that sinensetin mainly induces the proliferation of pulmonary fibroblasts *in vitro*.

**FIGURE 1:**
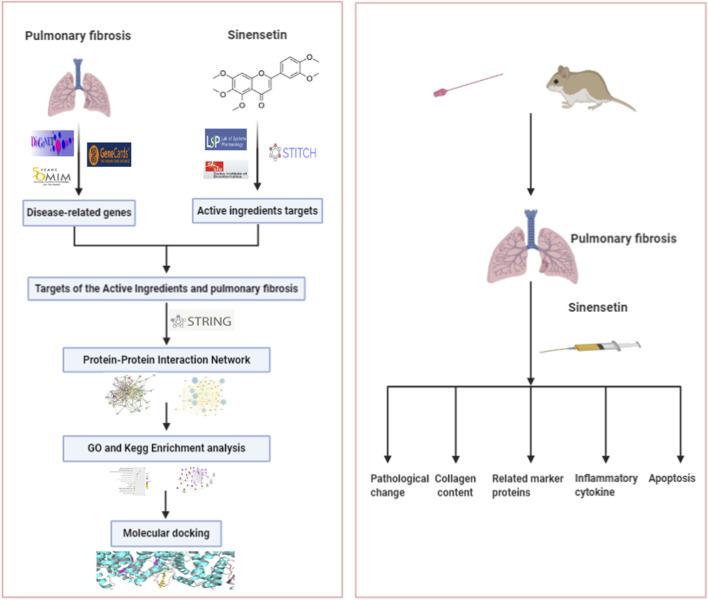
The study is organized based on network pharmacology and a PF mouse model.

Sinensetin is a type of polymethoxyflavone, distributed widely in the fruits and pericarps of citrus plants. It is also present in Citri Reticulatae Pericarpium, immature bitter orange, and other Chinese medical materials (Kang et al., 2015). The existing results showed that sinensetin has anti-inflammatory and anti-oxidant properties (Han Jie et al., 2021). Studies have found that sinensetin was able to mitigate the inflammatory response of LPS against mouse macrophages (J774) by inhibiting the activity of signal transducer and activator of transcription 1α (STAT1α) (Laavola et al., 2012). In addition, in an *in vitro* study, scholars concluded that sinensetin was able to attenuate H1N1 influenza virus–induced infection by inhibiting the NF-κB and MAPK signaling pathways (Li et al., 2020). *Via in vitro* and *in vivo* studies, Lam et al. (2012) concluded that sinensetin has good anti-angiogenic effects. However, the effects of sinensetin on PF remain poorly understood. This study aims to confirm the functional mechanism of sinensetin in PF based on a network pharmacology analysis and a PF mouse model. The technical process is shown in Figure 1.

## Materials and Methods

### Network Pharmacology

#### Prediction of the Functional Targets for Sinensetin

Related target genes for sinensetin were obtained from TCMSP (http://lsp.nwu.edu.cn/tcmsp.php), STITCH (http://stitch.embl.de/), SwissTargetPrediction (http://swisstargetprediction.ch/), and literature search.

#### Identification of Target Genes Related to PF

The databases used for the search of PF-related genes include GeneCards (http://www.genecards.org), OMIM (https://omim.org), and DisGeNET (http://www. disgenet.org). Duplicate genes were removed. The index words included “pulmonary fibrosis” or “lung fibrosis.”

#### Collection of Targets for PF Treatment with Sinensetin

Targets related to sinensetin were mapped to PF-related targets using R software. The intersections of both targets were considered potential targets for PF treatment with sinensetin.

#### Construction of a PPI Network

Gene targets for PF treatment with sinensetin were introduced into the STRING database to obtain PPI data, which were then imported into Cytoscape 3.6.0 to establish the protein–protein interaction (PPI) network.

#### KEGG Pathway Enrichment and GO Enrichment Analysis

The DAVID online tool (https://david.ncifcrf.gov/) was employed to merge the PPI network to obtain the core target proteins for KEGG pathway enrichment analysis and GO enrichment analysis. The resulting data were introduced into R software, and a bubble plot was constructed. The GO enrichment analysis includes biological process (BP), cellular component (CC), and molecular function (MF). Every gene was defined and described in several aspects. The ClueGO package in Cytoscape 3.6.0 software was used to establish the target–pathway network.

#### Molecular Docking

Molecular docking was conducted with sinensetin as the ligand and PI3K protein as the receptor using the AutoDock4 software. The Gasteiger method and LamarckianGA4.2 were employed to calculate the charge field and possible conformation for ligand–receptor binding, respectively. The docking between sinensetin and PI3K was tested using the binding energy of the reference ligand and the receptor, as well as the binding efficiency of the reference ligand and the ligand.

### Animal Experimentation

#### Reagents and Instruments

Sinensetin (Derick Company, China); bleomycin (BLM) hydrochloride (Novartis Pharma Co., Ltd., Switzerland); chloral hydrate (Sinopharm Chemical Reagent Co., Ltd., China); anti-collagens I and III, anti-vimentin, anti-α-SMA, anti-P-PI3K, anti-P-AKT, anti-PI3K, and anti-AKT (CST Company, United States) antibodies; mouse IL-6 kit; mouse hydroxyproline kit (Nanjing SenBeiJia Biotechnology Co., Ltd., China); and TUNEL apoptosis test kit (Beyotime Biotechnology Co., Ltd., China) were used in this study.

#### Test Animals and Groups

Healthy SPF C57BL/6J male mice (18–20 g, 6–8 weeks old) were purchased from Ji’nan Pengyue Animal Breeding Co., Ltd. (Ji’nan, China). The test animals were housed at room temperature in the pediatrics research institute of Nanjing University of Chinese Medicine for 1 week prior to the experiment. All animal studies were conducted in accordance with the guidelines and approved by the Ethics Committee of Nanjing University of Chinese Medicine.

Thirty mice were randomly assigned to one of five groups (six mice/group). The normal group received intragastric administration of physiological saline. The model group received the same treatment as the normal group. The high-, moderate-, and low-dosage groups were treated intragastrically with 200, 100, and 50 mg/kg/day of sinensetin, respectively. Two hours after dosing on the 28th day, the mice were killed through anesthetization. The lung tissues, serum, and bronchoalveolar lavage fluid (BALF) were collected. One part of lung tissues was fixed in 10% formalin, and the other part was frozen at −80°C prior to analysis.

#### Construction of the PF Model

A one-off intratracheal instillation of bleomycin was employed to construct the PF mouse model. After being fully anesthetized, the mice were placed on a board in a supine position, with the limbs and upper incisors fixed. The operator held the light in one hand to irradiate the neck of the mouse and gently pulled out the mouse’s tongue with the other hand using a pair of forceps. A blunt lumbar puncture needle was rapidly inserted into the trachea at the moment when the mouse inhaled. The prepared BLM solution was rapidly injected into the trachea with a syringe. After the modeling was finished, the mice were held in an upright position and rotated at a uniform speed to distribute BLM evenly in the lungs. Finally, the mice underwent thermal support and were returned to the cage.

#### Mouse Body Weight

The mice were weighed every week until the 28th day of the experiment.

#### Score of Alveolitis and PF

The lung tissues were fixed in 10% formalin, dehydrated with a gradient series of alcohol solutions, embedded in paraffin, sliced, and stained using HE and Masson. The degree of alveolitis and PF was scored according to the method of Ashcroft et al. (Ashcroft et al., 1988).

#### Measurement of Hydroxyproline Content in the Lung Tissue

The level of hydroxyproline in the lung tissues was determined by ELISA. Fifty milligrams of lung tissues were homogenized, and the supernatant was aspirated for use. The assay kit was removed from the refrigerator and rewarmed at room temperature for 30 min. The concentrated wash buffer was diluted with double-distilled water. The blank control, sample, and standard wells were set up on the plate according to the requirements of the kit, and the corresponding reagents were added. The standard curve was plotted according to the measured OD values, and the measured concentrations of the samples in each well were calculated according to the curve equation; these concentration values were then multiplied by the dilution factors to obtain the final concentrations.

#### Measurement of IL-6 in Serum and BALF

The samples were removed from the −80°C freezer and defrosted at room temperature. After the standard substances were diluted, the samples were loaded and washed, the enzyme was added, the solution was incubated, and the color was developed following the user’s manual for the IL-6 ELISA kit. Then, the linear regression equation of the standard curve was calculated based on concentrations of the standard and corresponding absorbance values. Concentrations of the samples were calculated based on the absorbance values of samples and the regression equation. The final concentration of samples = measured concentration × dilution factor.

#### Expression of Collagens I and III, Vimentin, and *α*-SMA in Lung Tissues

Paraffin sections were sectioned at a thickness of approximately 4 µm. After dewaxing and hydration, 3% hydrogen peroxide was added to the sections at room temperature for 15 min for blocking the activity of endogenous peroxidases. Goat serum was added in a dropwise manner for blocking non-specific binding, and the sections were incubated at room temperature for 30 min. The serum was then removed, and the sections were incubated overnight at 4°C with the primary antibodies. On the next day, the slides were washed, treated with the secondary antibodies and the color development solution (separately), and then re-stained with hematoxylin; after dehydration and blocking, the slides were observed under a microscope. The appearance of brownish yellow areas under light microscopy (×200 magnification) was considered a positive result. The integrated optical density (IOD)/area was measured using Image-Pro Plus 7.0 software. The optical density was first corrected, and then, the IOD and area values were measured separately.

#### TUNEL Staining of Lung Tissues

The paraffin-embedded tissue sections were dewaxed using xylene and dehydrated using a graded series of ethanol solutions; then, the sections were dried by shaking, and the tissues were circled using a PAP pen. Proteinase K was added for antigen retrieval, after which the tissue sections were covered by adding a lysis working buffer. After this, the reaction solution was added separately, and the nuclei were re-stained with DAPI. Finally, the sections were sealed with antifade mounting medium. The fluorescence intensity of the samples was analyzed using Image-Pro Plus 7.0 software.

#### Expression of Related Proteins by Western Blotting

The lung tissues were lysed using the RIPA buffer containing proteinase inhibitors. The protein content was measured, separated by 8–12% SDS/PAGE, and then electroblotted to a PVDF membrane. Non-specific antigens were incubated in 5% BSA with the membrane for 1.5 h. Then, the membrane was incubated overnight at 4°C in a 10-time diluted solution of corresponding antibodies. The membrane was washed in 1% TBST for three times for 10 min at room temperature (RT). Next, the secondary antibody (1:2000) was added, and the mixture was incubated for 1 h at RT. The membrane was washed with TBST for three times for 10 min and then exposed to a color-developing reagent in dark. The gray values were analyzed using Image-Pro Plus 7.0 software.

#### Data Analysis

SPSS 22.0 software was used for data analysis. The quantitative metrological data are expressed as mean ± SD. Between-group differences were tested with ANOVA. If the difference was significant, the least significant difference t test was used for multiple comparisons. Statistical significance was set as *p* < 0.05.

## Results

### Prediction of Targets for PF Treatment With Sinensetin

**FIGURE 2:**
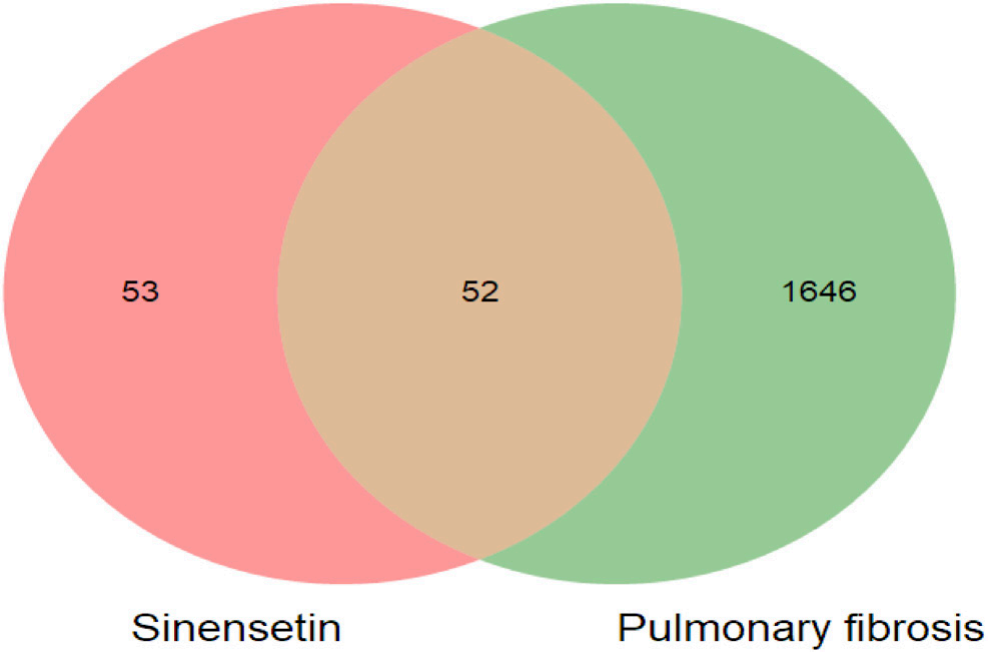
Target screening of sinensetin to ameliorate PF. Venn diagram of candidate targets for PF treatment with sinensetin.

The targets for sinensetin were screened using the TCMSP, STITCH, and SwissTargetPrediction databases, with a total of 105 drug targets obtained. In total, 1,698 gene targets highly related to PF were obtained, and the duplicates were removed using the GeneCards, OMIM, and DisGeNET databases. The intersection of the drug targets and PF-related targets was considered to represent the candidate targets for PF treatment with sinensetin; in total, 52 targets were obtained (Table 1). The intersection containing these 52 targets is presented in a Venn diagram (Figure 2 and Supplementary File S1).

**TABLE 1:** Potential targets for PF treatment with sinensetin.

Gene name	Gene ID	Target
ABCB1	5,243	P-glycoprotein 1
ABCC1	4,363	Multidrug resistance–associated protein 1
ACHE	43	Acetylcholinesterase
ADORA1	134	Adenosine A1 receptor
ALK	238	ALK tyrosine kinase receptor
ALOX5	240	Arachidonate 5-lipoxygenase
APEX1	328	DNA-(apurinic or apyrimidinic site) lyase
ARG1	383	Arginase-1 (by homology)
BCL2L1	598	Apoptosis regulator Bcl-X
BMP4	652	Bone morphogenetic protein 4 (by homology)
CA4	762	Carbonic anhydrase IV
CCNB1	891	Cyclin-dependent kinase 1/cyclin B
CDK1	983	Cyclin-dependent kinase 1/cyclin B
CDK2	1,017	Cyclin-dependent kinase 2
CFTR	1,080	Cystic fibrosis transmembrane conductance regulator
CXCR1	3,577	Interleukin-8 receptor A
CYP1A1	1,543	Cytochrome family 1 subfamily a member 1
CYP1A2	1,544	Cytochrome family 1 subfamily a member 2
CYP1B1	1,545	Cytochrome family 1 subfamily B member 1
EGFR	1956	Epidermal growth factor receptor erbB1
ESR1	2099	Estrogen receptor alpha
F2	2,147	Thrombin
FLT3	2,322	Tyrosine-protein kinase receptor FLT3
GSK3B	2,932	Glycogen synthase kinase-3 beta
IGF1R	3,480	Insulin-like growth factor I receptor
INSR	3,643	Insulin receptor
KDR	3,791	Vascular endothelial growth factor receptor 2
KIT	3,815	Stem cell growth factor receptor
MAPK1	5,594	MAP kinase ERK2
MET	4,233	Hepatocyte growth factor receptor
MMP12	4,321	Matrix metalloproteinase 12
MMP13	4,322	Matrix metalloproteinase 13
MMP2	4,313	Matrix metalloproteinase 2
MMP9	4,318	Matrix metalloproteinase 9
MPO	4,353	Myeloperoxidase
NOS2	4,843	Nitric oxide synthase, inducible
NOX4	50,507	NADPH oxidase 4
PARP1	142	Poly [ADP-ribose] polymerase-1
PIK3CG	5,294	PI3-kinase p110-gamma subunit
PLA2G2A	5,320	Phospholipase A2 group IIA
PLG	5,340	Plasminogen
PLK1	5,347	Serine/threonine-protein kinase PLK1
PTGS2	5,743	Cyclooxygenase-2
PTK2	5,747	Focal adhesion kinase 1
SRC	6,714	Tyrosine-protein kinase SRC
SYK	6,850	Tyrosine-protein kinase SYK
TERT	7,015	Telomerase reverse transcriptase
TOP1	7,150	DNA topoisomerase I (by homology)
TOP2A	7,153	DNA topoisomerase II alpha
TTR	7,276	Transthyretin
TYR	7,299	Tyrosinase
XDH	7,498	Xanthine dehydrogenase

### Construction of the PPI Network

**FIGURE 3:**
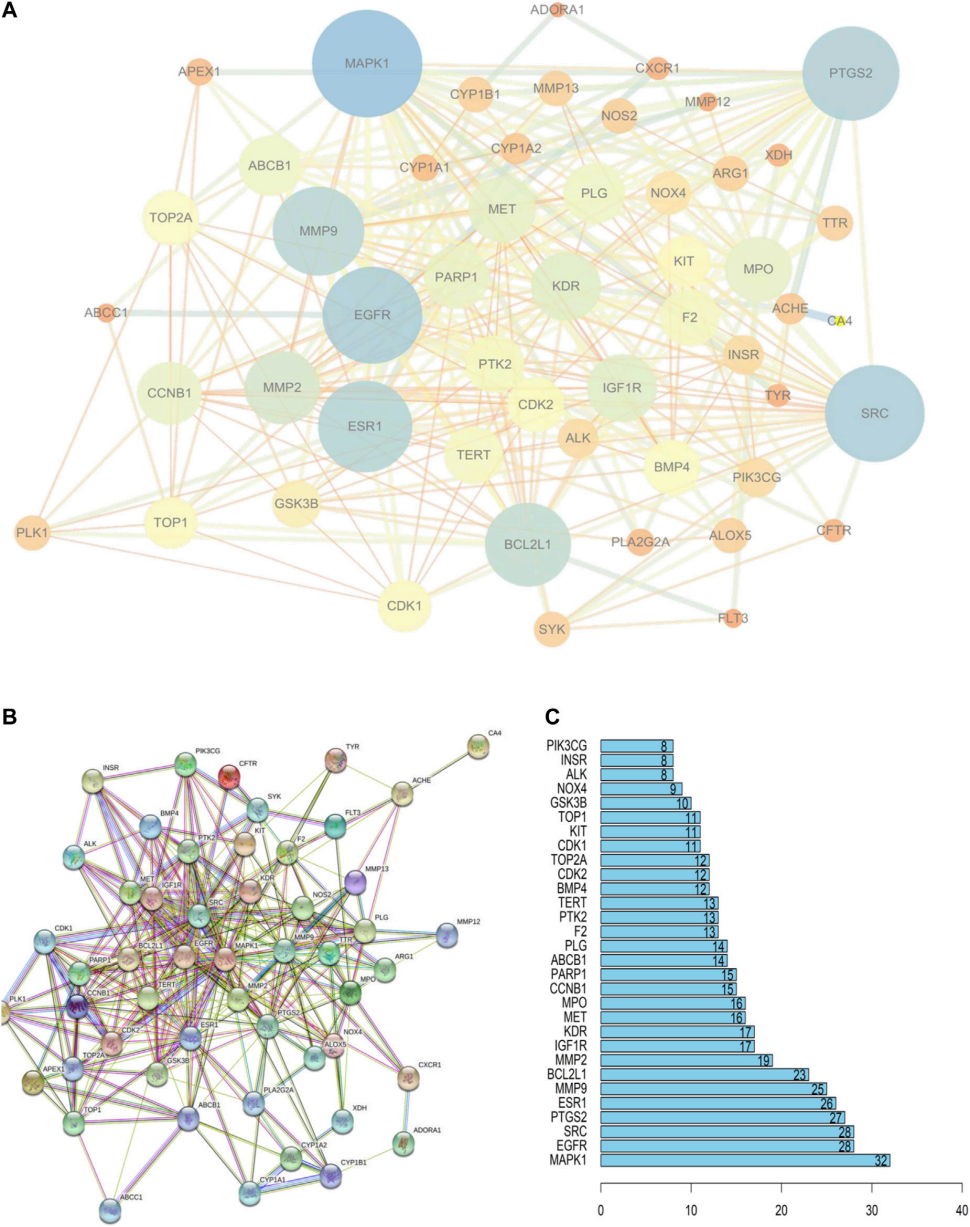
PPI network of PF treatment with sinensetin. **(A)** STRING data were exported using Cytoscape 3.6.1 software. **(B)** PPI network analysis. **(C)** The top 30 nodes are likely key proteins in the interaction.

These 52 targets were introduced into STRING to obtain the PPI network (Figure 3B). STRING data were exported to draw a network diagram using Cytoscape 3.6.1 software (Figure 3A). The results from R showed that MAPK1, EGFR, SRC, and PTGS2 were potentially important targets in the network (Figure 3C).

### GO Enrichment Analysis

**FIGURE 4:**
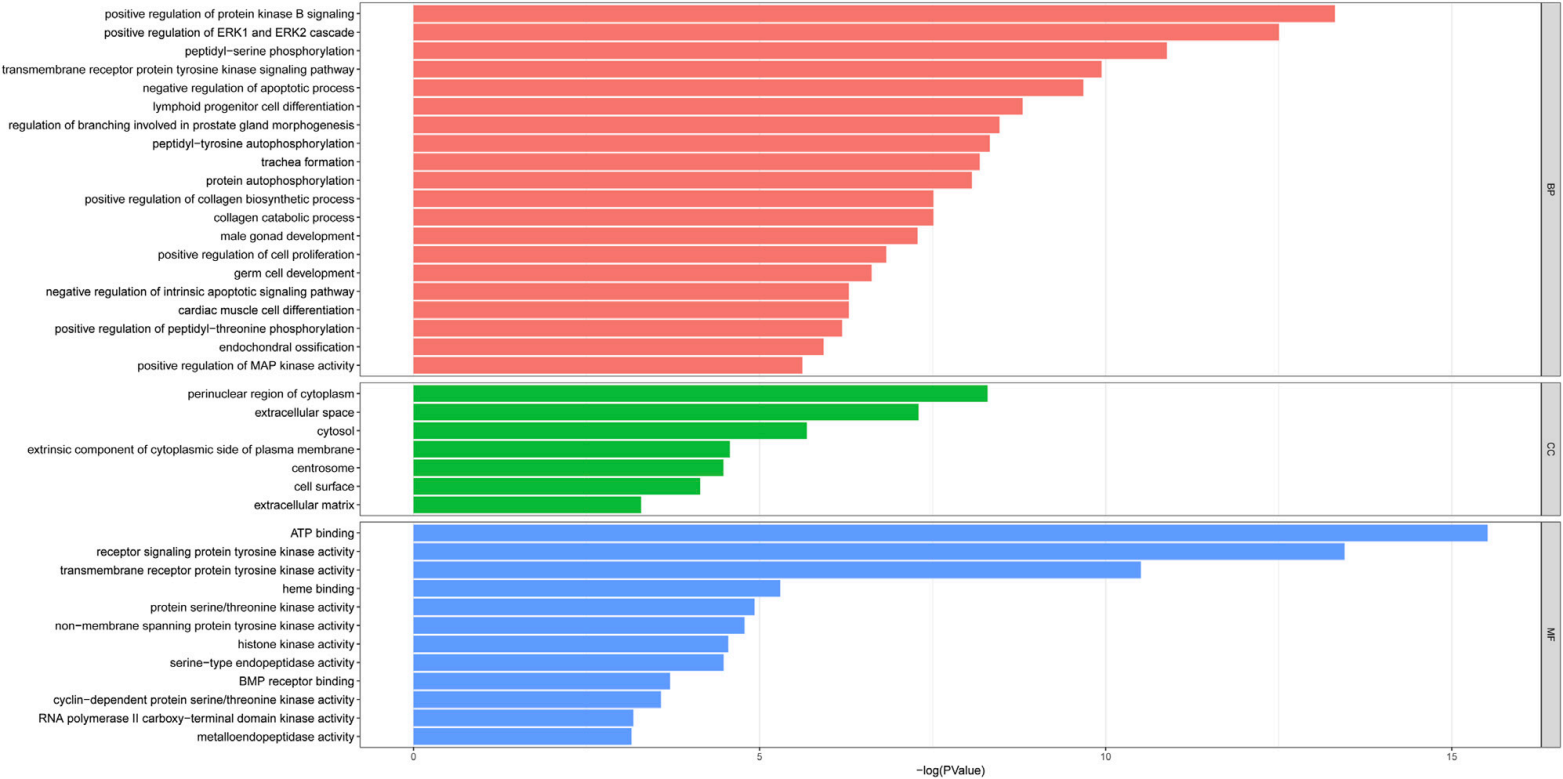
GO enrichment of 52 potential targets. The top enriched terms covering the BP, CC, and MF are presented.

To predict the functional mechanism underlying the treatment of PF with sinensetin, we used the DAVID website to perform GO enrichment analysis for the BP of these 52 potential targets. As shown in Figure 4, the BP terms mainly included cell apoptosis, cell proliferation, and protein phosphorylation. The CC mainly included the region of cytoplasm, extracellular space, and cytosol, and the MF mainly included ATP binding, protein kinase activity, and heme binding (Figure 4).

### KEGG Pathway Enrichment Analysis

**FIGURE 5:**
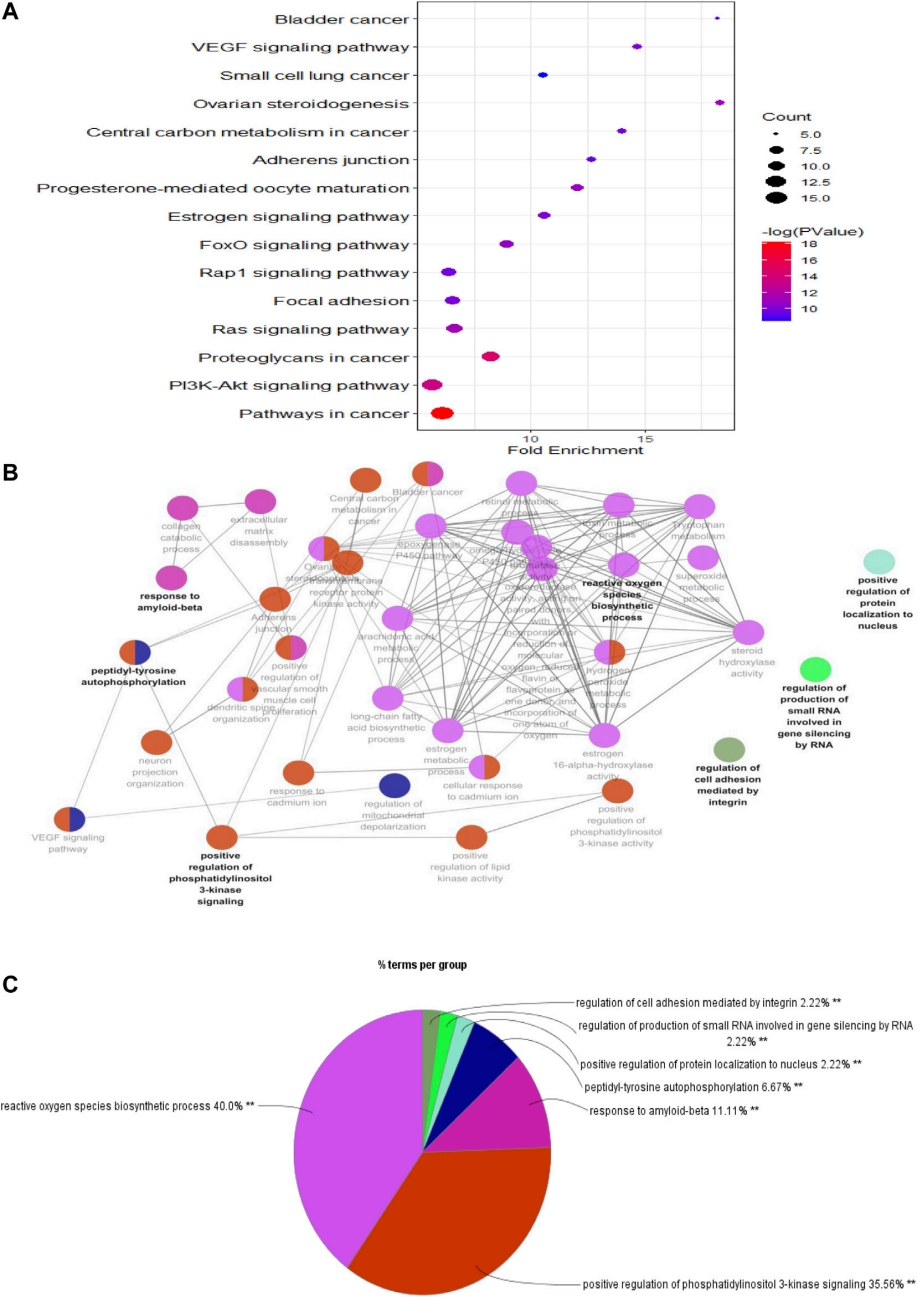
KEGG pathway enrichment analysis. **(A)** Bubble chart showing the top 15 KEGG pathway enrichment results. **(B)** Pathway relationship network using Cytoscape software. **(C)** Percentage of each main pathway count for the signaling pathways.

To predict the potential signaling pathways involved in PF treatment with sinensetin, we input these 52 common targets into the DAVID website for KEGG pathway analysis. The results showed that PI3K and many other signaling pathways were affected by the treatment of PF with sinensetin. Subsequently, we introduced these 52 common functional targets into Cytoscape to construct the target–pathway network using the ClueGO plugin. This analysis also revealed that multiple signaling pathways, including the PI3K pathway, were probably affected by the treatment of PF with sinensetin (Figure 5).

### Molecular Docking Analysis

**FIGURE 6:**
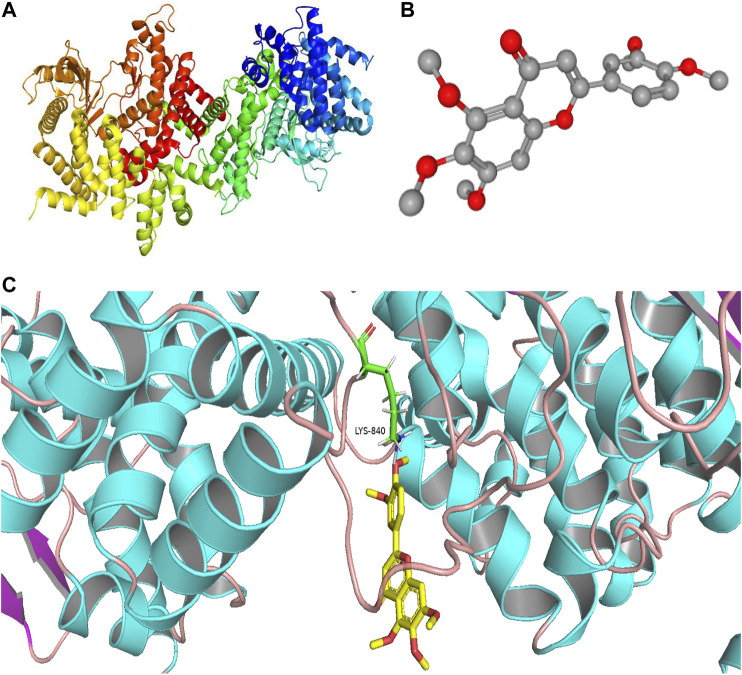
Molecular docking of sinensetin to PI3K. **(A)** Molecular structures of PI3K protein. **(B)** Chemical structure of sinensetin. **(C)** Molecular docking simulation between sinensetin and PI3K.

We found that the PI3K signaling pathway most likely had a critical effect on the treatment of PF with sinensetin. We tested the molecular docking of sinensetin with the PI3K protein using the AutoDock4 software. The results showed that sinensetin could fit well into several binding sites of the PI3K protein and that the binding energy at the LYS-840 site was -5.5 kcal/ml (Figure 6).

### Mouse Body Weight Change

**FIGURE 7:**
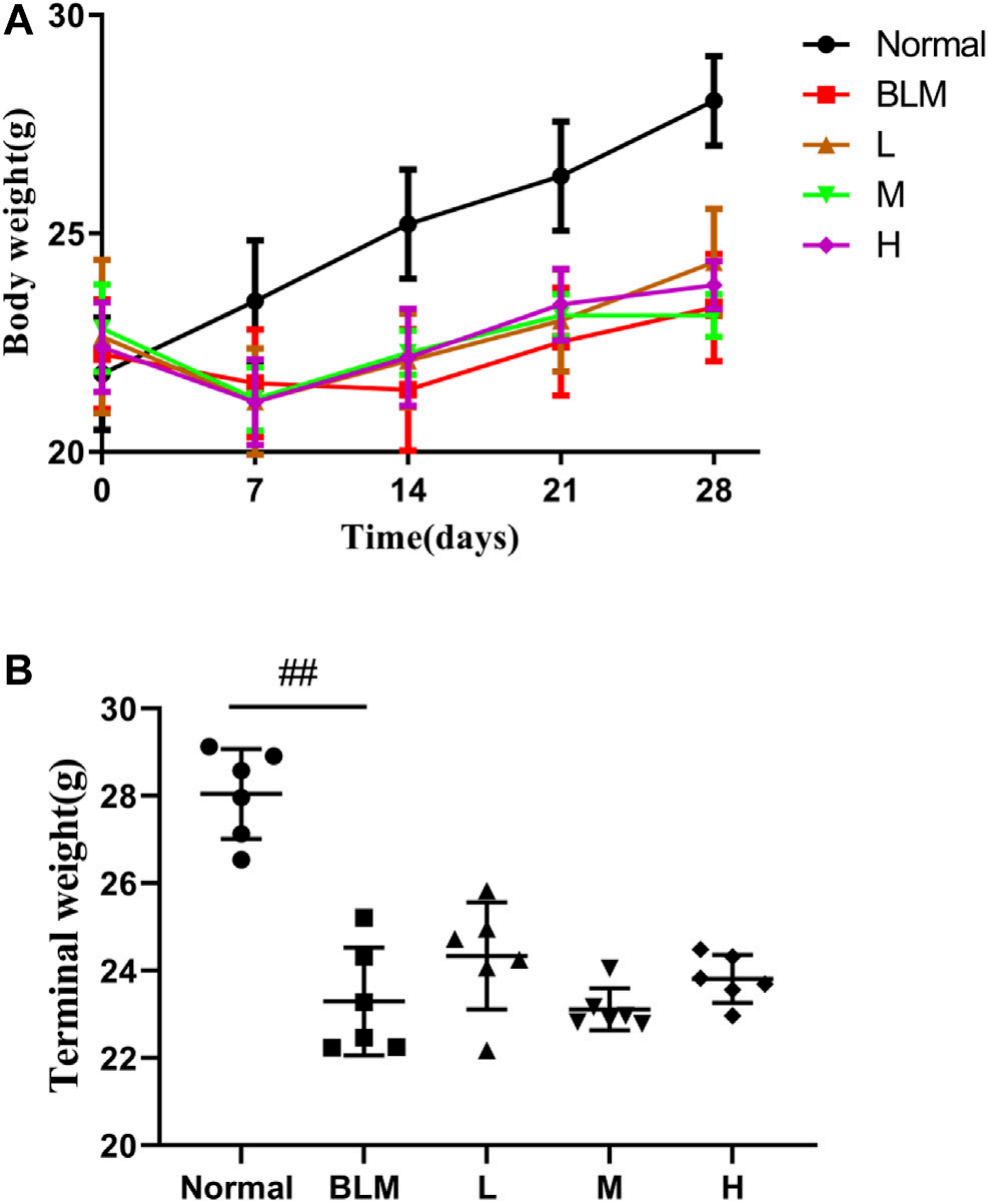
Body weight changes of mice after treatment with sinensetin. **(A)** Average body weights at different time points. **(B)** Final body weights in the different groups. Normal: normal group; BLM: model group; L: low-dosage sinensetin group; M: moderate-dosage sinensetin group; H: high-dosage sinensetin group. Data reported in the figures are mean ± SD, n = 6 in each group. ##*p* < 0.01 vs. the normal group.

The results showed that mouse body weight increased continuously in the normal group but reduced in all other groups in the week after the model was established (Figure 7A). However, more than 1 week after model construction, the mouse body weight increased. The final body weight in the model group was reduced profoundly relative to that in the normal group. Although the mice in the high-, moderate-, and low-dosage sinensetin groups gained some body weights, no statistical differences between the interventions were observed (Figure 7B).

### Pathological Change

**FIGURE 8:**
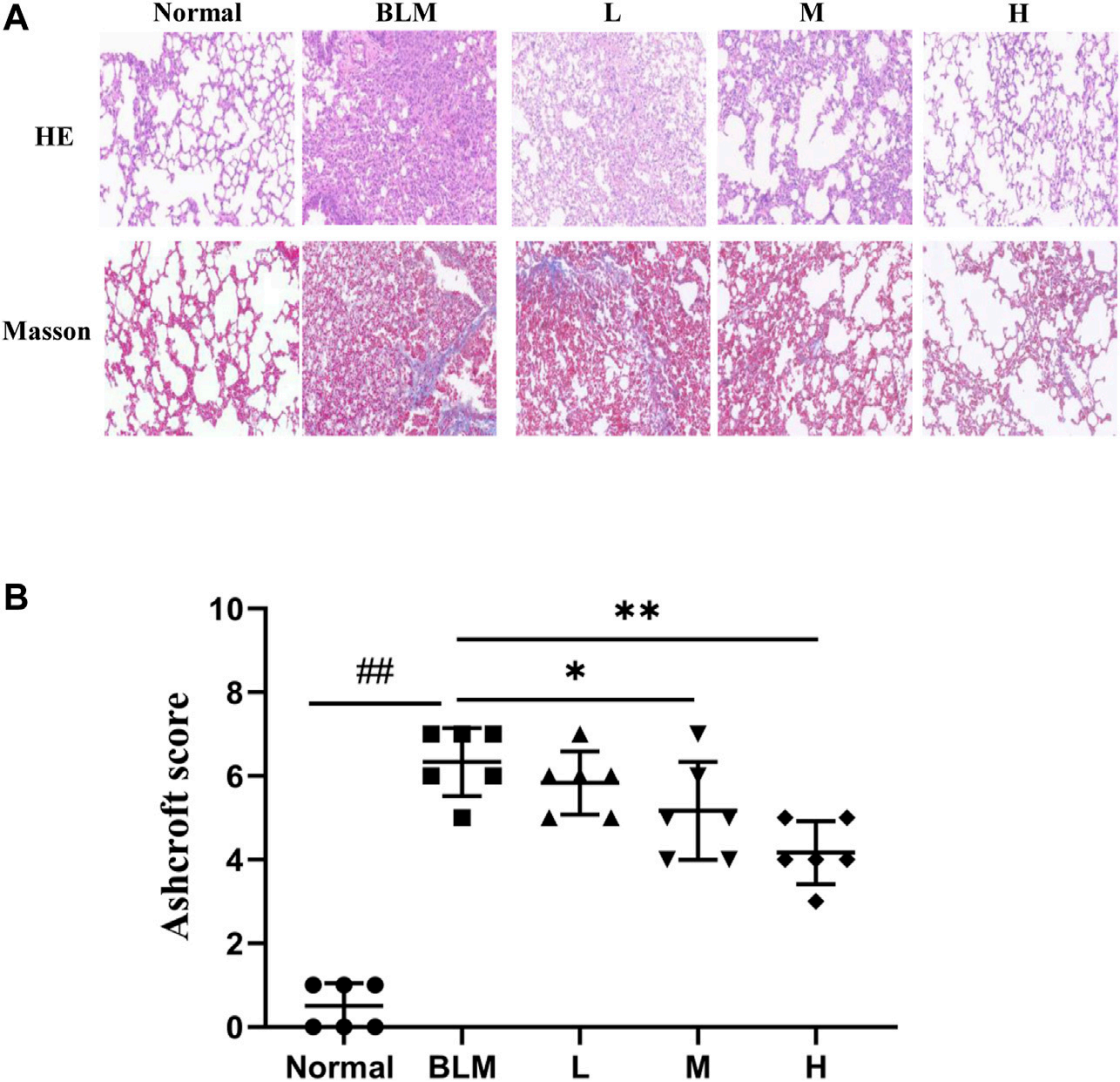
Pulmonary pathological changes of sinensetin treatment. **(A)** HE and Masson’s trichrome staining of lung tissues, magnification ×200. **(B)** Ashcroft score in each group. Normal: normal group; BLM: model group; L: low-dosage sinensetin group; M: moderate-dosage sinensetin group; H: high-dosage sinensetin group. Data reported in the figures are mean ± SD, n = 6 in each group. ^##^ *p* < 0.01 vs. the normal group; *p < 0.05 and **p < 0.01 as compared to the BLM group.

The results of HE staining showed that the normal group had a normal lung tissue structure, whereas the model group showed a significantly widened alveolar interval, collapsed pulmonary alveolus fusion, and disorganized structure, accompanied by massive inflammatory infiltration. Significant amelioration was observed in the high-dosage sinensetin group as compared to the model group. The results of Masson staining showed a small amount of blue collagen deposition in lung tissues of the normal group. In contrast, a massive blue collagen deposition was observed in the model group. After interventions with sinensetin, collagen deposition in the lung tissues of the model mice was reduced to a certain extent, and a marked reduction was found in the high-dosage sinensetin group (Figure 8A).

As a further evaluation of the pathological degree of PF, we scored alveolitis and PF using the Ashcroft method based on the results of HE staining and Masson staining. The results suggested that the scores for Ashcroft and PF in the high- and moderate-dosage sinensetin groups were significantly reduced as compared to those in the model group, whereas no significant difference was observed in the low-dosage sinensetin group (Figure 8B).

### Collagen Contents

**FIGURE 9:**
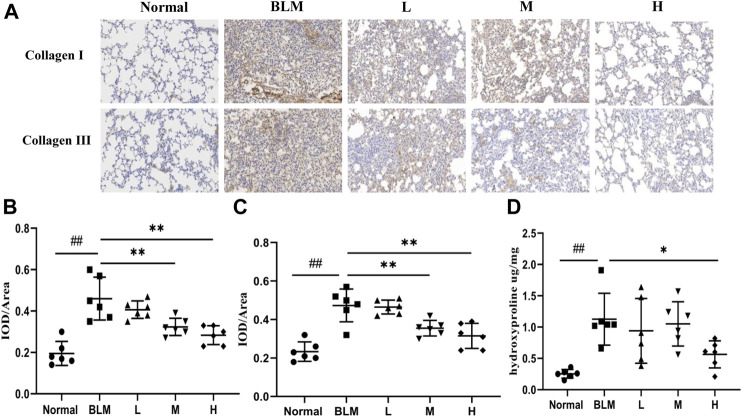
Effects of sinensetin treatment on the deposition of collagen in lung tissues. **(A)** Immunohistochemical analysis of collagen I and collagen III (200x). **(B)** Immunohistochemical analysis of collagen I. **(C)** Immunohistochemical analysis of collagen III. **(D)** Expressions of hydroxyproline in lung tissues. Normal: normal group; BLM: model group; L: low-dosage sinensetin group; M: moderate-dosage sinensetin group; H: high-dosage sinensetin group. Data reported in the figures are mean ± SD, n = 6 in each group. ^##^ *p* < 0.01 vs. the normal group; *p < 0.05 and **p < 0.01 as compared to the BLM group.

To test the effect of sinensetin on collagen deposition in the lung tissues, we assayed the expression of collagens I and III using immunohistochemical analyses and quantified the content of hydroxyproline using ELISA. The results of the immunohistochemical analyses showed that the content of type I and type III collagens in mouse lung tissues in the model group was markedly higher than that in the normal group. In contrast, the content of both collagens in lung tissues after sinensetin interventions was reduced to a certain extent as compared to that in the model group, and the greatest reduction was found in the high-dosage sinensetin group (Figures 9A–C). The hydroxyproline content in the lung tissues in the model group was markedly higher as compared to that in the normal group, and its content was reduced significantly after interventions with high-dosage sinensetin (Figure 9D).

### Expression of Marker Proteins in Lung Tissues

**FIGURE 10:**
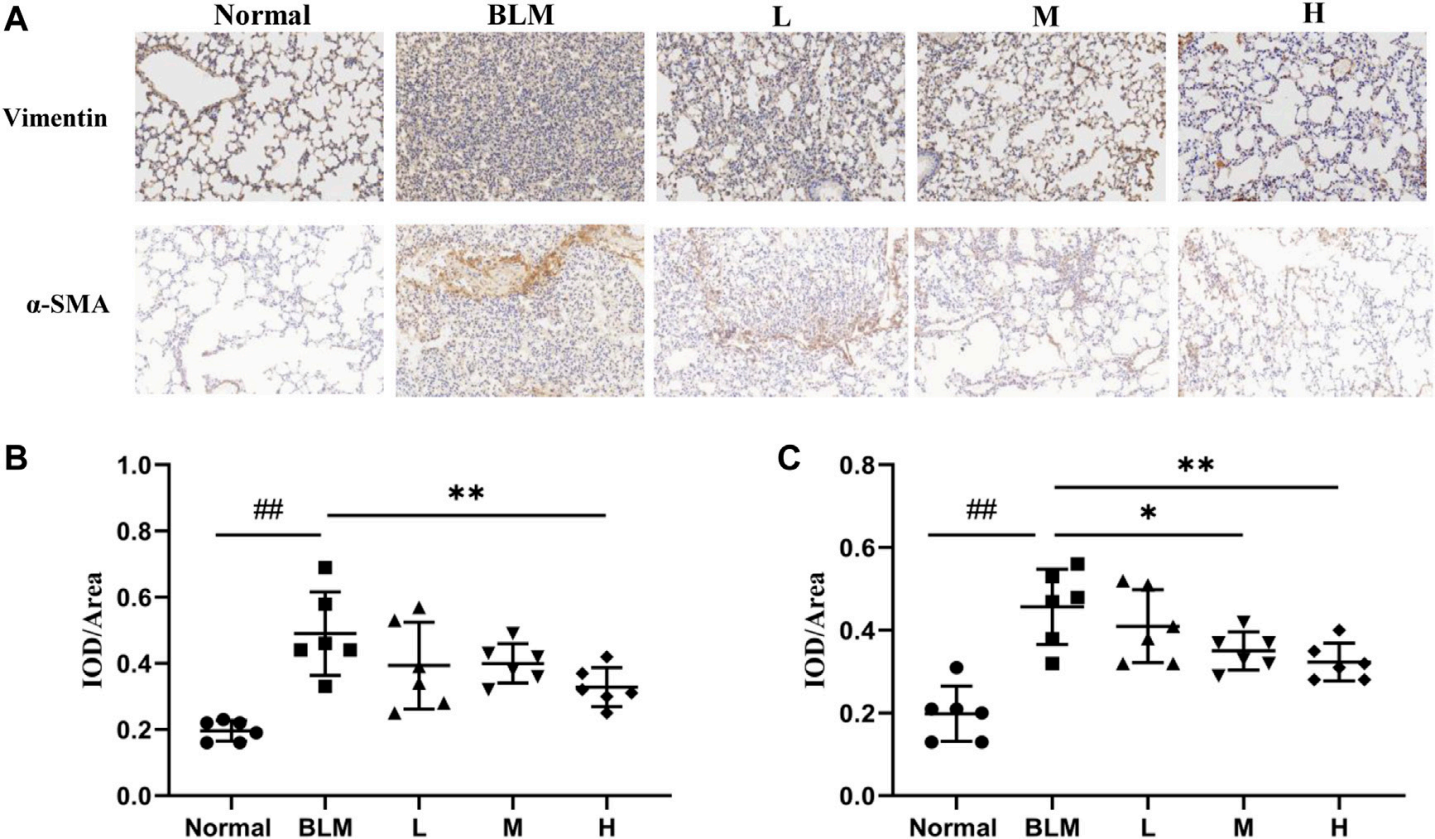
Effects of sinensetin on the expression of marker proteins. **(A)** Immunohistochemical assay of vimentin and *α*-SMA in lung tissues (200x). **(B)** Immunohistochemical analysis of vimentin. **(C)** Immunohistochemical analysis of *α*-SMA. Normal: normal group; BLM: model group; L: low-dosage sinensetin group; M: moderate-dosage sinensetin group; H: high-dosage sinensetin group. Data reported in the figures are mean ± SD, n = 6 in each group. ^##^ *p* < 0.01 vs. the normal group; *p < 0.05 and **p < 0.01 as compared to the BLM group.

The basic pathological change in PF is a mass proliferation of pulmonary fibroblasts and their conversion into myofibroblasts. Vimentin and *α*-SMA are the characteristic marker proteins for pulmonary fibroblasts and myofibroblasts, respectively. We, therefore, used the expression of these two proteins in lung tissues to estimate the extent of PF. The results of the immunohistochemical analyses showed that the expression of vimentin was significantly upregulated in the model group than in the normal group. In contrast, the expression of vimentin was markedly downregulated in the high-dosage sinensetin group than in the model group (Figures 10A,B).

The expression of *α*-SMA was markedly enhanced in the model group than in the normal group (Figures 10A,C), whereas its expression in lung tissues in the high- and moderate-dosage sinensetin groups was significantly lower than that in the model group. This suggested that successful model construction was accompanied by the mass proliferation of pulmonary fibroblasts and myofibroblasts and that sinensetin administration could markedly reduce the proliferation of pulmonary fibroblasts and myofibroblasts.

### Levels of Inflammatory Cytokines

**FIGURE 11:**
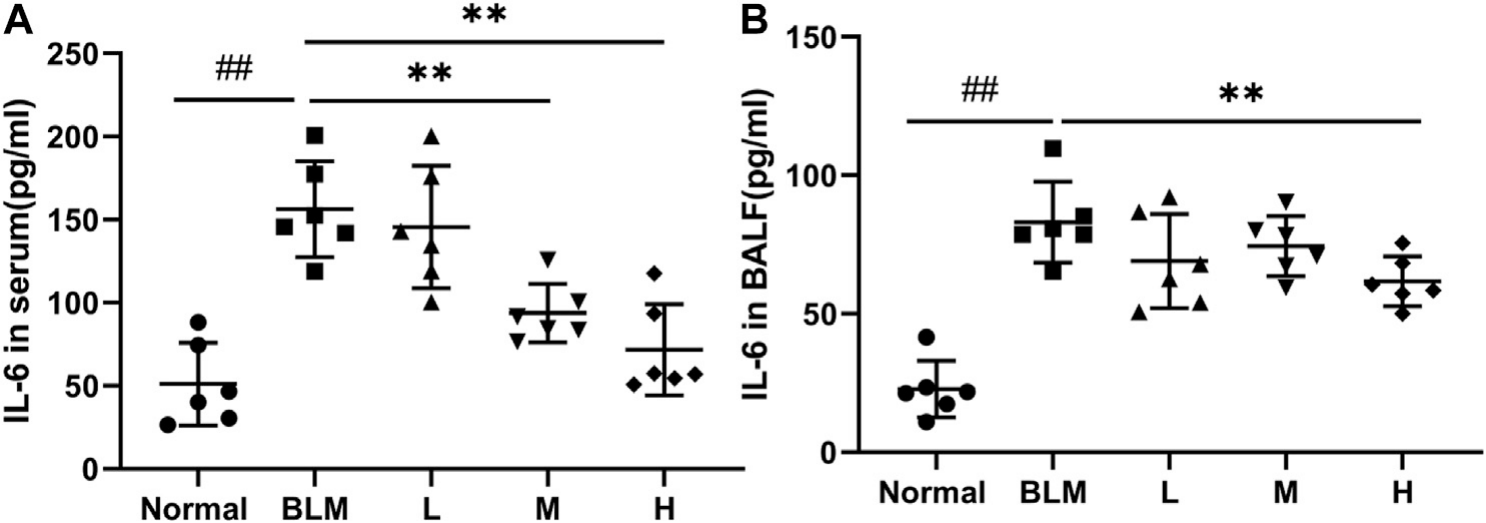
Effects of sinensetin on the plasma levels of inflammatory markers. **(A)** IL-6 production in the serum was quantified using the ELISA method. **(B)** IL-6 production in the BALF was measured using the ELISA method. Normal: normal group; BLM: model group; L: low-dosage sinensetin group; M: moderate-dosage sinensetin group; H: high-dosage sinensetin group. Data reported in the figures are mean ± SD, n = 6 in each group. ^##^ *p* < 0.01 vs. the normal group; **p < 0.01 vs. the BLM group.

Inflammatory reactions have played an important part in the occurrence and development of PF; notably, IL-6 is a key cytokine. To test whether sinensetin could inhibit the inflammatory reaction during the progression of PF, we measured the changes in IL-6 in the BALF and serum. As shown in Figure 11, the moderate- and high-dosage sinensetin groups showed markedly reduced levels of serum IL-6. High-dosage sinensetin also effectively reduced the level of IL-6 in the BALF.

### Cell Apoptosis in Lung Tissues

**FIGURE 12:**
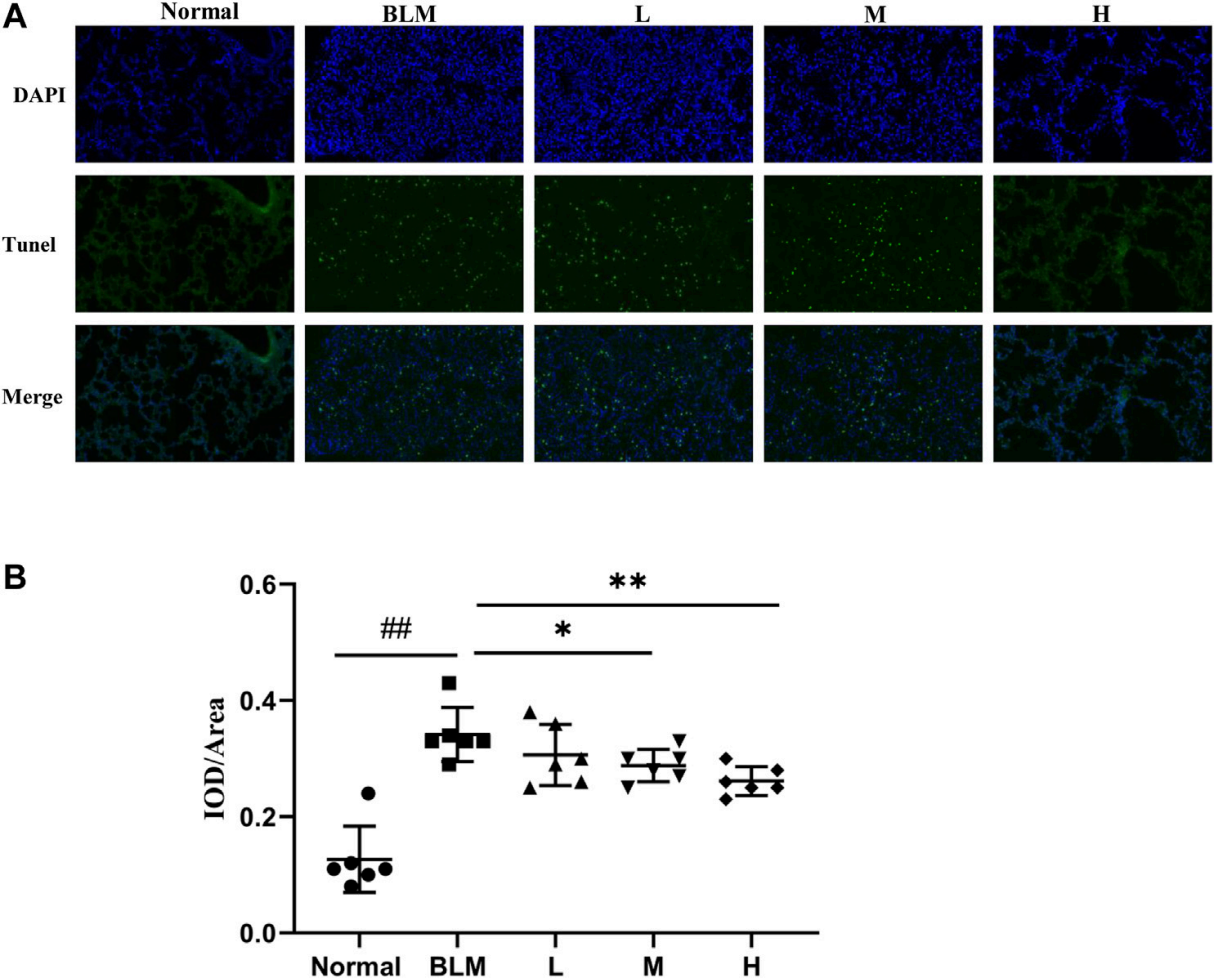
Effects of sinensetin on apoptosis in pulmonary tissues. **(A)** Apoptosis was visualized by the TUNEL assay (200x). **(B)** Immunofluorescence analysis of apoptosis. Normal: normal group; BLM: model group; L: low-dosage sinensetin group; M: moderate-dosage sinensetin group; H: high-dosage sinensetin group. Data reported in the figures are mean ± SD, n = 6 in each group. ^##^ *p* < 0.01 vs. the normal group; *p < 0.05 and **p < 0.01 as compared to the BLM group.

The extent of apoptotic cells can reflect the degree of pathological changes in PF. Based on our network pharmacological analysis, the effect of sinensetin on PF was considered to be related to the regulation of cell apoptosis. To test this hypothesis, we used the TUNEL method to measure the degree of cell apoptosis in the lung tissues. As apoptosis usually occurs in the nuclei, we quantified apoptosis through the superposition of blue and green fluorescence. The results of the TUNEL test suggested that the proportion of apoptotic cells in the mouse model group was significantly more than that in the normal group (Figure 12). In contrast, the proportions in the lung tissues in the high- and moderate-dosage sinensetin groups were significantly reduced as compared to that in the model group.

### Expression of PI3K and AKT in Lung Tissues

**FIGURE 13:**
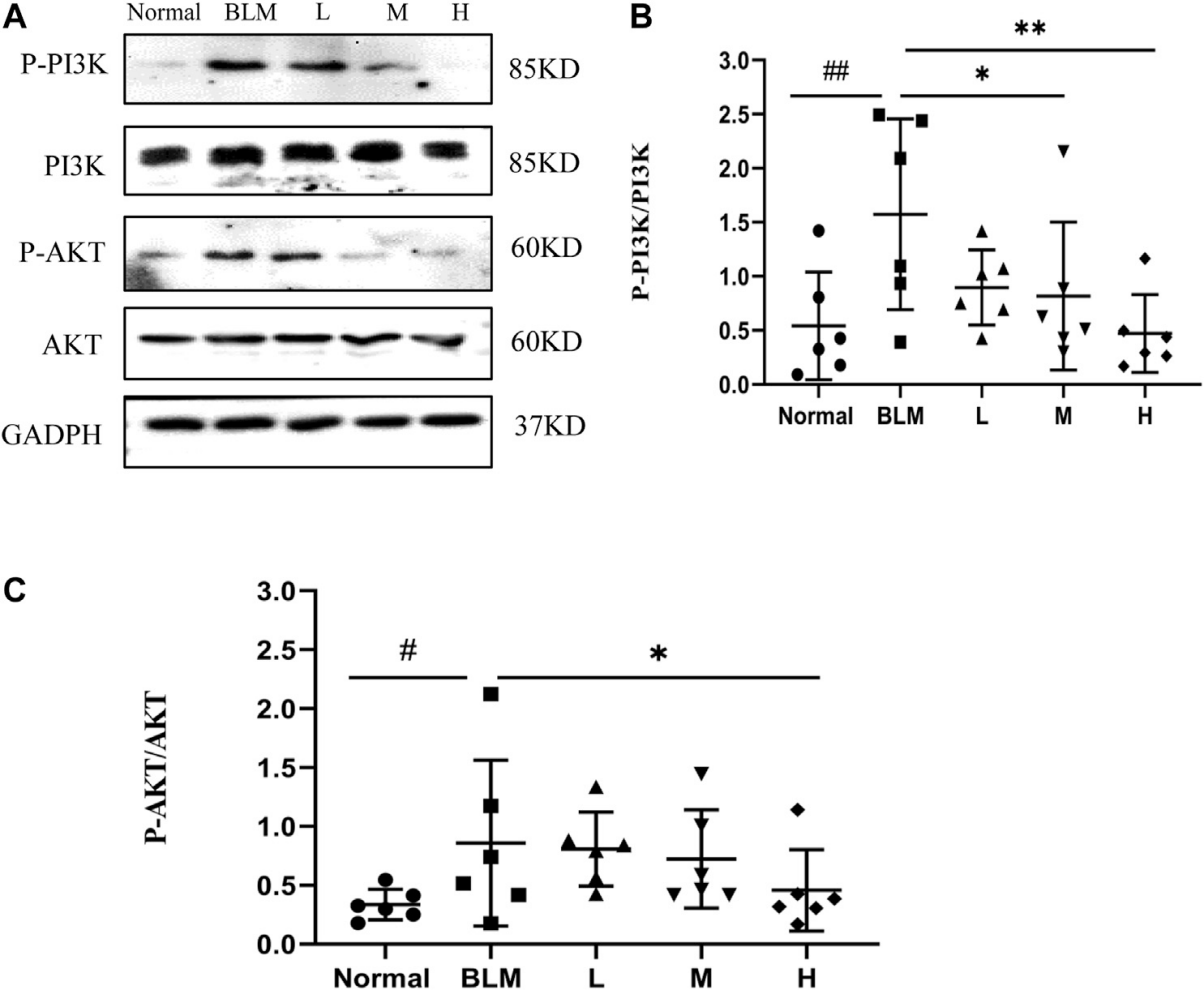
Effects of sinensetin on the expression of P-PI3K and P-AKT in pulmonary tissues. **(A)** The expressions of P-PI3K and P-AKT in the pulmonary tissues were recorded by western blot. **(B)** Quantitative result of P-PI3K. **(C)** Quantitative result of P-AKT. Normal: normal group; BLM: model group; L: low-dosage sinensetin group; M: moderate-dosage sinensetin group; H: high-dosage sinensetin group. Data reported in the figures are mean ± SD, n = 3 in each group. ^#^ *p* < 0.05 and ^##^ *p* < 0.01 vs. the normal group; *p < 0.05 and **p < 0.01 as compared to the BLM group.

Based on our KEGG enrichment analysis, the effect of sinensetin on PF was likely to be mediated through the PI3K/AKT signaling pathway. To test this hypothesis, we measured the transcript levels of P-PI3K, P-AKT, PI3K, and AKT in mouse lung tissues by western blotting. The results showed that the expressions of P-PI3K and P-AKT were increased markedly after bleomycin injection, whereas their expression was reduced, to a certain extent, after sinensetin interventions (Figure 13). Moreover, these changes were much clearer in the high-dosage sinensetin group.

## Discussion

Network pharmacology analysis is used to construct, simulate, and analyze drugs, proteins, genes, and other biochemical information using computer technology (Zeng et al., 2019). It can consider the multiple effects of drugs through a network, helping to reduce the toxic effects of drugs, increase their curative efficiency, improve treatment success rates, and reduce the costs of drug research and development (Guo et al., 2019). In the future, network pharmacology will become the predominant method used for drug research and development (Zhang et al., 2019). In the present study, network pharmacology analysis revealed that there were 52 potential targets for PF treatment with sinensetin that were enriched in multiple biological pathways and signaling pathways. Based on GO analysis and KEGG pathway analysis, we predicted that cellular apoptosis and the PI3K/AKT signaling pathway probably had critical roles in sinensetin-mediated PF treatment.

In this paper, we constructed a PF mouse model and treated the mice with sinensetin to test this hypothesis. To define the protective role of sinensetin in mice with PF, we examined the pathology of the lung tissues using HE staining and Masson staining; furthermore, we estimated the extent of PF by scoring PF and alveolitis. As suggested by the structural and pathological changes in lung tissues, sinensetin can, to a certain extent, delay the development of PF in mice. The basic pathological feature of PF is the mass deposition of extracellular matrix components (Deng et al., 2020; Mou and Mou, 2020). The results showed that the expressions of type I and type III collagens in the lung tissues of PF mice were increased profoundly after bleomycin model construction and that the expressions of both types of collagens were reduced markedly after sinensetin interventions, especially in the high-dosage sinensetin group. The results for hydroxyproline also followed this trend. Therefore, it was shown that sinensetin could inhibit pulmonary collagen deposition and delay the development of PF.

In addition, we further investigated whether sinensetin could delay the progression of pulmonary fibrosis by examining the expression of inflammatory factors and lung fibroblast markers. The results showed that, after sinensetin intervention, IL-6 levels in mouse lung tissues and BALF were significantly reduced, while vimentin and *α*-SMA expressions decreased significantly. These results suggest that sinensetin effectively delayed the progression of pulmonary fibrosis by reducing collagen deposition, reducing the inflammatory response, and inhibiting lung fibroblast proliferation.

Given the in-depth investigations into the pathogenesis of PF, evidence has confirmed that cell apoptosis, especially of alveolar epithelial cells, is an important event during the initiation and development of PF (Zhu et al., 2019). The degree of cell apoptosis in lung tissues reflects, to a certain extent, the degree of pathological changes in PF (Johnson et al., 2020). It was proven that alveolar epithelial apoptosis occurs in relatively normal lung tissues at the peripheral focus in patients with idiopathic PF (Xie et al., 2017). Alveolar epithelial apoptosis in patients with idiopathic PF can lead directly to alveolar collapse and accelerate the advancement of PF (Korfei et al., 2008). These findings support the important role of alveolar epithelial apoptosis in the early stage of PF (Bhandary et al., 2012). In the present study, the proportion of apoptotic cells in the model group was markedly more than that in the normal group. By contrast, the proportion of apoptotic cells in lung tissues of PF mice was significantly reduced by sinensetin interventions. This suggests a possible role of sinensetin in PF, which was consistent with the GO analysis of our network pharmacology study.

It has been reported that cell apoptosis is enhanced in bleomycin-induced PF mice. Further studies found that this apoptosis was probably related to the PI3K/AKT signaling pathway (Cully, 2020). In the present study, P-PI3K and P-AKT were significantly upregulated in the lung tissues of the model group than those in the normal group. In contrast, after sinensetin administration, the expressions of both markers in lung tissues of the model group were reduced as compared to that in the normal group. It was speculated that the curative effect of sinensetin on PF was probably related to the downregulation of P-PI3K and P-AKT, which was consistent with the predictions of network pharmacology.

However, there are still limitations in this study that need to be addressed through further research. Firstly, although this study concluded that sinensetin could delay the progression of pulmonary fibrosis, it is not known whether sinensetin can reduce the damage to the alveolar epithelial cells or inhibit the proliferation of lung fibroblasts and, if both mechanisms exist, which mechanism is dominant. Further cellular experiments are needed to confirm these aspects. Secondly, this study failed to perform a thorough investigation of the sinensetin-mediated regulation of apoptosis *via* the PI3K/AKT signaling pathway, which needs to be validated by performing subsequent *in vivo* and *in vitro* experiments using PI3K and AKT knockouts. Finally, although we have learned that apoptosis is inhibited by sinensetin, three pathways are known to regulate apoptosis: the membrane receptor pathway, the mitochondrial pathway, and the endoplasmic reticulum pathway; which of these three apoptotic pathways is predominant requires further investigation.

## Conclusion

This paper predicted the potential targets for PF interventions by sinensetin based on network pharmacology. The results showed that the functional mechanism for sinensetin-mediated PF treatment was probably related to cell apoptosis regulation and PI3K/AKT signaling pathway regulation. A mouse model of PF was constructed using a one-off intratracheal bleomycin instillation, and the mice were then treated with sinensetin. The results showed that sinensetin could be used to treat PF and that this activity was probably related to the orchestration of the PI3K/AKT signaling pathway and cell apoptosis. Our findings of network pharmacology were highly in agreement with those of animal tests. The results of the present study would supply a reference for PF treatment.

## Data Availability

The original contributions presented in the study are included in the article/Supplementary Material, and further inquiries can be directed to the corresponding authors.
