# Supplementary material for: Exploring the Mechanism Whereby Sinensetin Delays the Progression of Pulmonary Fibrosis Based on Network Pharmacology and Pulmonary Fibrosis Models
Source: Front Pharmacol. 2021 Jun 18;12:693061. doi: 10.3389/fphar.2021.693061 (PMC8249588; doi:10.3389/fphar.2021.693061)
Supplement: Supplementary file 1 [file Presentation1.PPTX]

## Slide 1
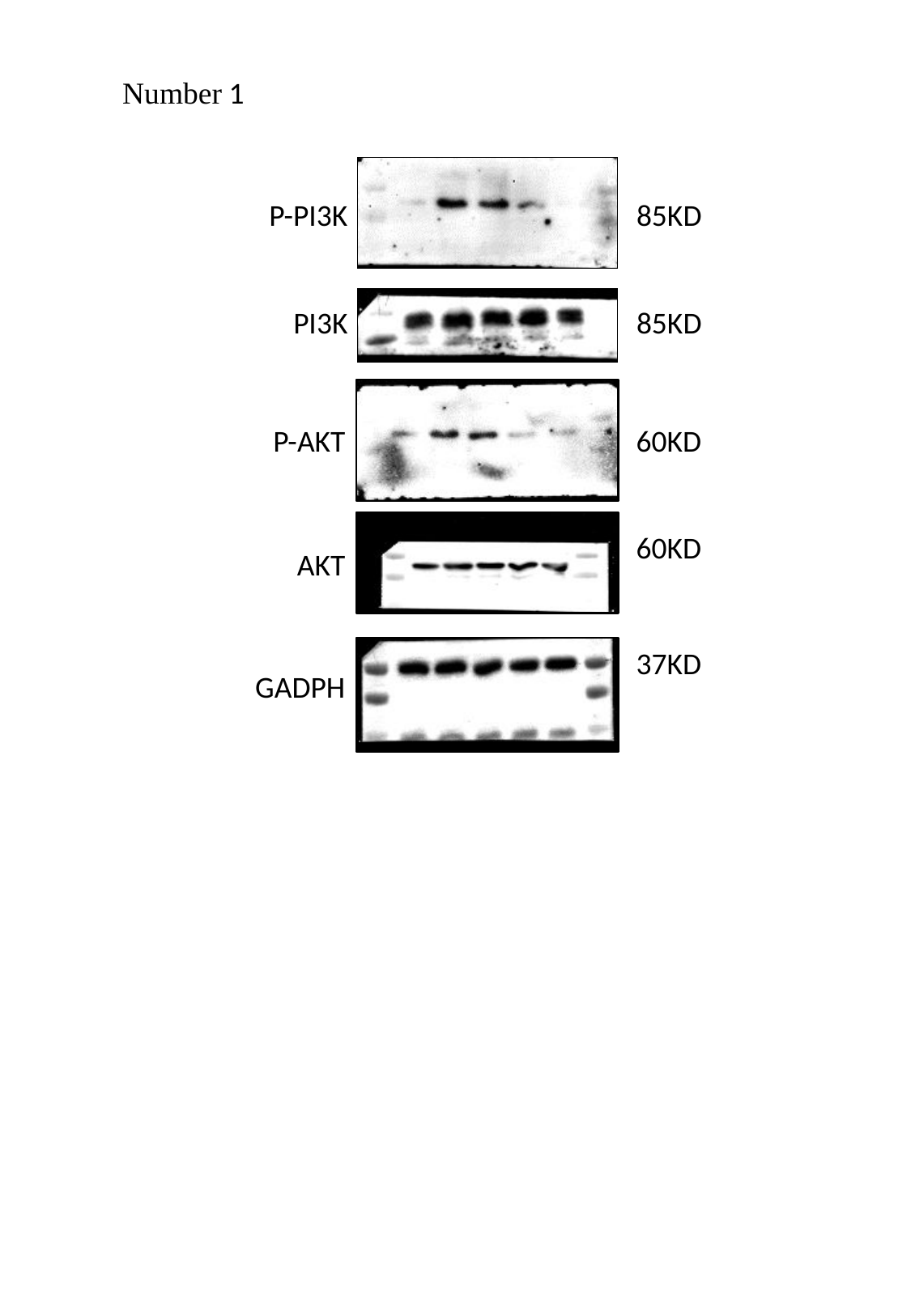

Number 1
P-PI3K
85KD
PI3K
85KD
P-AKT
60KD
60KD
AKT
37KD
GADPH

## Slide 2
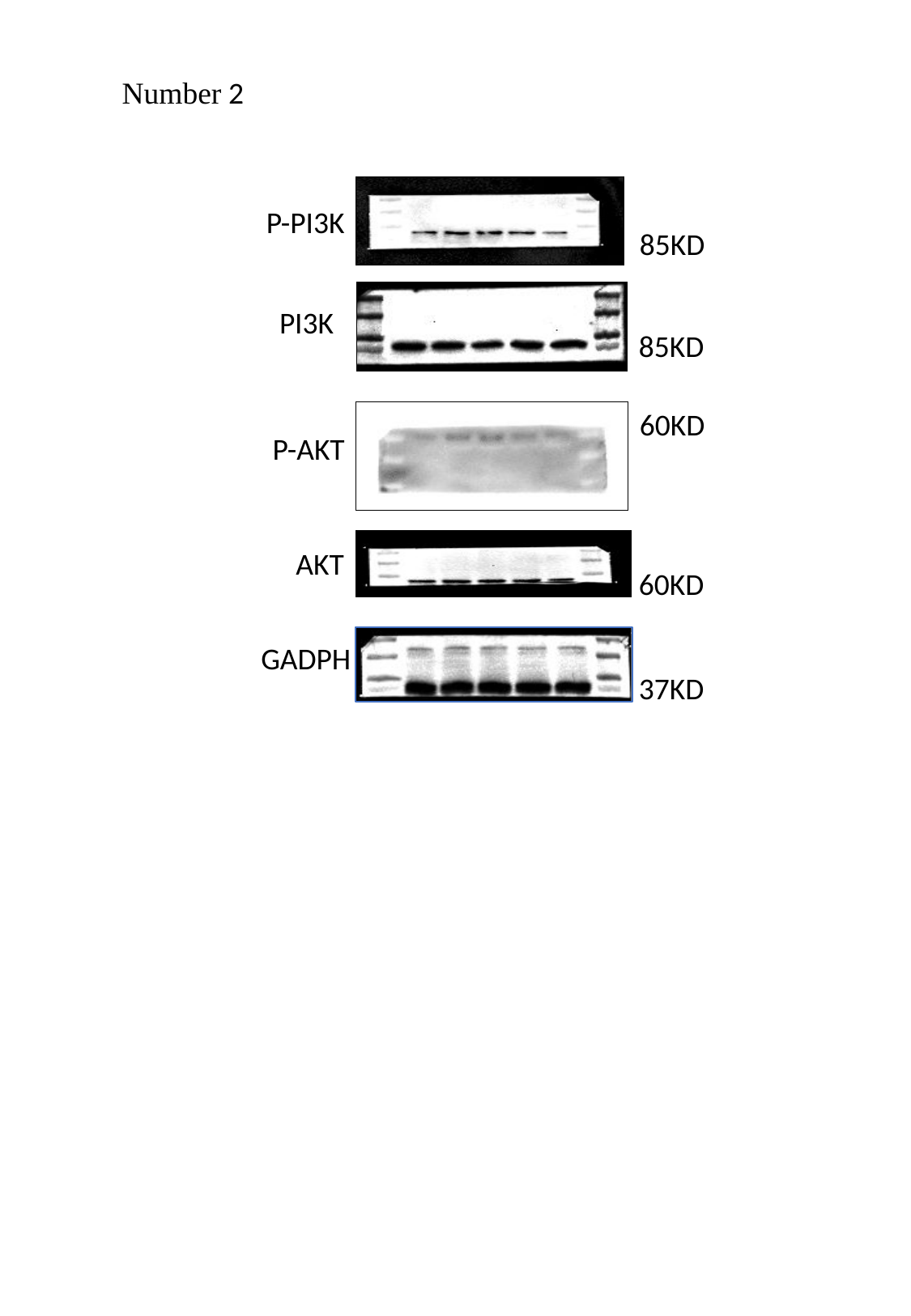

Number 2
P-PI3K
85KD
PI3K
85KD
60KD
P-AKT
AKT
60KD
GADPH
37KD

## Slide 3
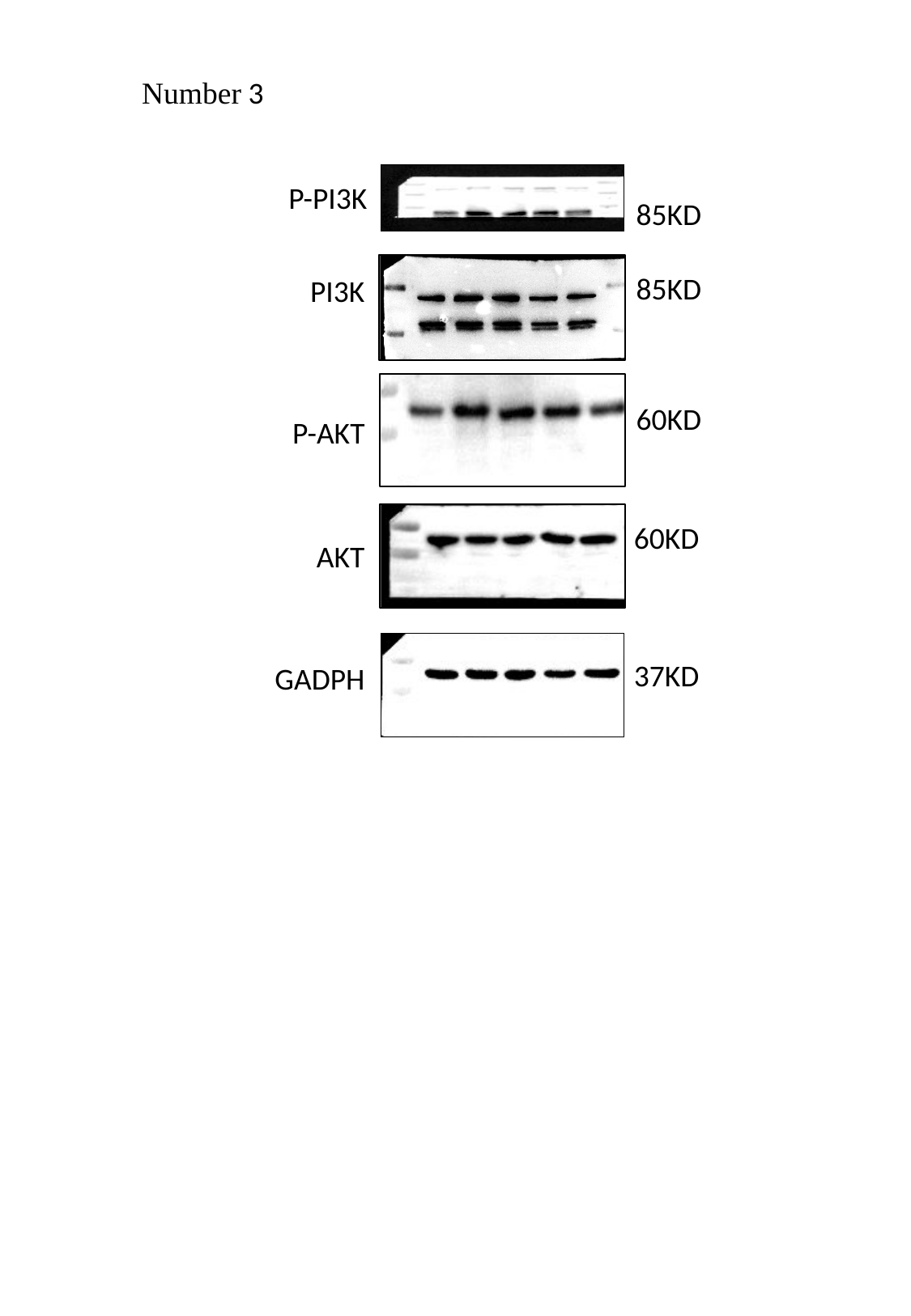

Number 3
P-PI3K
85KD
85KD
PI3K
60KD
P-AKT
60KD
AKT
37KD
GADPH

## Slide 4
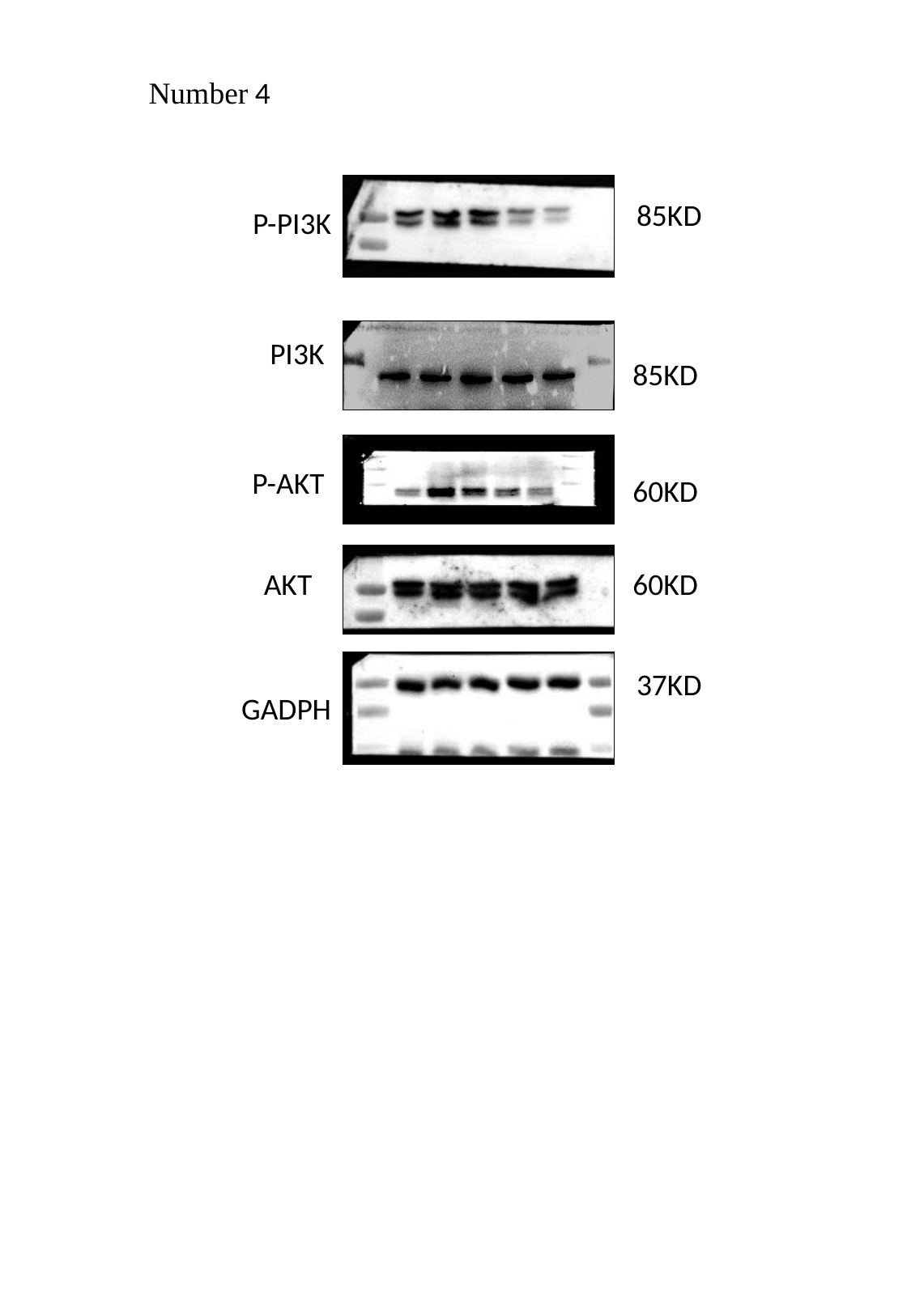

Number 4
85KD
P-PI3K
PI3K
85KD
P-AKT
60KD
AKT
60KD
37KD
GADPH

## Slide 5
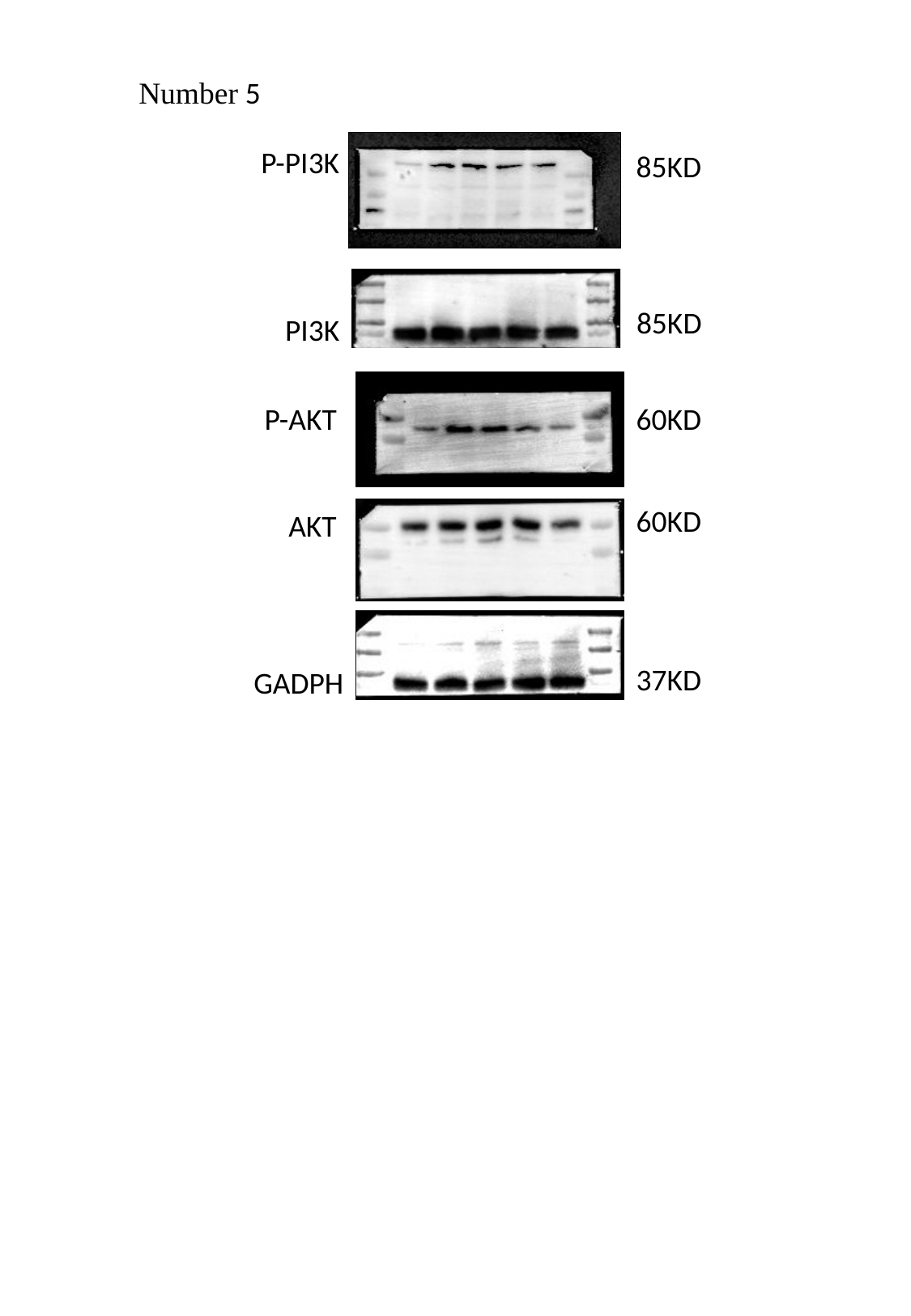

Number 5
P-PI3K
85KD
85KD
PI3K
60KD
P-AKT
60KD
AKT
37KD
GADPH

## Slide 6
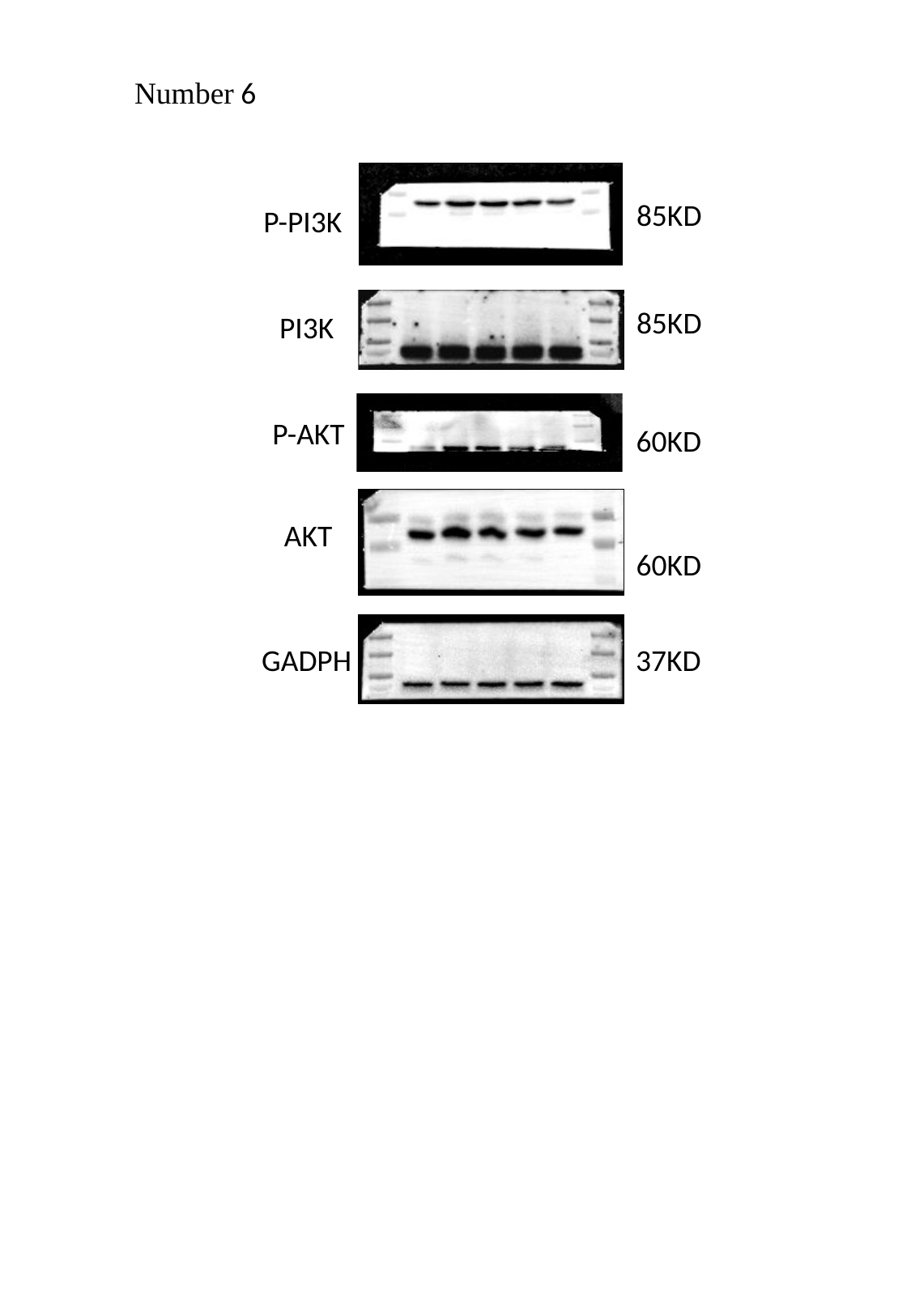

Number 6
85KD
P-PI3K
85KD
PI3K
P-AKT
60KD
AKT
60KD
GADPH
37KD
