# Supplementary figures and images for: Exploring the Mechanism Whereby Sinensetin Delays the Progression of Pulmonary Fibrosis Based on Network Pharmacology and Pulmonary Fibrosis Models
Source: Front Pharmacol. 2021 Jun 18;12:693061. doi: 10.3389/fphar.2021.693061 (PMC8249588; doi:10.3389/fphar.2021.693061)

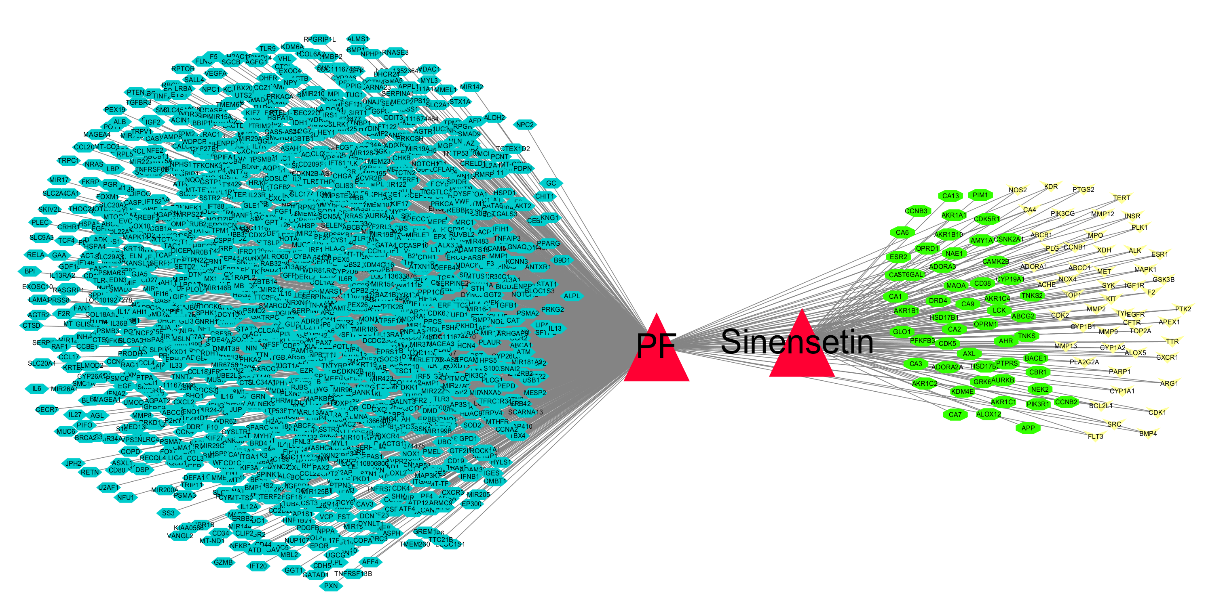


Supplementary files-1：The network of PF treatment with sinensetin.

Supplement: Supplementary file 2 [file DataSheet1.docx]
